# Predictors of Mortality After Liver Transplantation for Acute Liver Failure: A Multicenter Cohort Study

**DOI:** 10.1155/cjgh/5051145

**Published:** 2026-08-31

**Authors:** Micheli Fortunato Domingos, Júlio Cezar Uili Coelho, Eduardo José Brommelstroet Ramos, Jorge Eduardo Fouto Matias, Fabio Silveira, João Eduardo Leal Nicoluzzi, Nertan Luiz Tefilli, Julio Cesar Wiederkehr, Abdo Imad El Tawil, Gabriel Kindermann de Farias

**Affiliations:** ^1^ Department of Surgery and Transplantation, Nossa Senhora das Graças Hospital, Curitiba, State of Paraná, Brazil; ^2^ Department of Surgery and Transplantation, Clinical Hospital, Federal University of Paraná, Curitiba, State of Paraná, Brazil, ufpr.br; ^3^ Department of Transplantation, Rocio Hospital, Campo Largo, State of Paraná, Brazil; ^4^ Department of Transplantation, Angelina Caron Hospital, Campina Grande do Sul, State of Paraná, Brazil; ^5^ Department of Transplantation, São Vicente Hospital, Curitiba, State of Paraná, Brazil; ^6^ Department of Transplantation, Mackenzie Evangelical University Hospital, Curitiba, State of Paraná, Brazil; ^7^ Department of Transplantation, Santa Isabel Hospital, Blumenau, State of Santa Catarina, Brazil, hsi.org.br

**Keywords:** acute liver failure, liver transplantation, mortality predictors

## Abstract

**Introduction:**

Acute liver failure (ALF) is a rare and life‐threatening condition characterized by rapid deterioration of liver function and often requires urgent liver transplantation (LT). This study aimed to evaluate donor and recipient factors associated with 30‐day mortality after LT for ALF.

**Methods:**

Medical records of patients who underwent LT for ALF at seven Brazilian transplant centers were reviewed. Patient data were obtained from electronic medical records. The primary outcome was 30‐day mortality after LT.

**Results:**

Ninety patients who underwent LT for ALF were included. Thirty‐day mortality occurred in 46 of 90 patients (51%). In multivariable analysis, higher pretransplant serum creatinine levels (OR 3.49, 95% CI 1.69–8.72; *p* = 0.003) and the need for mechanical ventilation prior to transplantation (OR 5.46, 95% CI 1.21–28.9; *p* = 0.033) were independently associated with early mortality. Donor age ≥ 50 years and pretransplant vasoactive drug use were associated with mortality in univariate analysis.

**Conclusion:**

Higher pretransplant serum creatinine levels and the need for mechanical ventilation were independently associated with 30‐day mortality, whereas donor age ≥ 50 years and pretransplant vasoactive drug use were associated with mortality only in the univariate analysis. These findings support improved pretransplant risk stratification and perioperative management in patients undergoing LT for ALF.

## 1. Introduction

Acute liver failure (ALF) is a rare, life‐threatening, and potentially reversible condition characterized by the rapid onset of hepatocellular dysfunction, resulting in severe liver impairment, coagulopathy, and hepatic encephalopathy in patients without preexisting chronic liver disease [1–3]. ALF is associated with high mortality in the absence of liver transplantation (LT), which remains the only definitive therapeutic option for patients with poor prognosis [4, 5]. Mortality rates without transplantation may reach up to 50%–80%, highlighting the critical importance of early referral and timely organ allocation [6]. Due to its rapid progression and high risk of death, ALF is considered a condition of highest priority for LT, defined by the development of hepatic encephalopathy within 8 weeks after the onset of jaundice in patients without underlying liver disease who meet established prognostic criteria such as the King’s College or Clichy criteria [7].

Although ALF accounts for a small proportion of liver transplant indications, it remains a clinically significant condition due to its high mortality and the urgent need for transplantation. Prospective studies have demonstrated poor outcomes in patients who do not undergo LT [8]. Registry data from the Organ Procurement and Transplantation Network (OPTN), the Scientific Registry of Transplant Recipients (SRTR), and the European Liver Transplant Registry (ELTR) indicate that ALF accounts for approximately 2%–7% of LTs worldwide [9, 10]. In Brazil, detailed registry data on ALF are not available from the Associação Brasileira de Transplante de Órgãos (ABTO); however, a multicenter study including 12 liver transplant centers reported that ALF accounted for 5.4% of 6030 liver transplants performed between 2006 and 2015 [11].

Despite advances in intensive care, surgical techniques, and perioperative management, outcomes after LT for ALF remain inferior to those observed in patients transplanted for chronic liver disease [3–5, 12]. This is largely related to the greater clinical severity, hemodynamic instability, and the emergent nature of transplantation in this population. Early prospective studies have highlighted the severe clinical course and high mortality associated with advanced ALF [13].

Data on ALF in Latin America remain limited [11, 14, 15]. A multicenter Brazilian study demonstrated that drug‐induced liver injury (DILI) represents one of the leading causes of ALF in the country, highlighting regional differences in etiology [11]. Similarly, a multicenter Argentine cohort reported clinical outcomes after LT for ALF, emphasizing the need for regional analyses of prognostic factors [15]. The King’s College Criteria remain the most widely used tool to guide the indication for urgent transplantation in ALF [16]; however, they were designed to predict survival without transplantation and provide limited information regarding posttransplant outcomes.

Therefore, identifying clinical factors associated with early mortality after LT in patients with ALF remains an important challenge. Improved understanding of these factors may help optimize perioperative management and improve patient selection in this critically ill population. The aim of the present study was to evaluate the impact of donor and recipient characteristics on 30‐day mortality after LT for ALF and to identify factors independently associated with early posttransplant mortality in a multicenter Brazilian cohort.

## 2. Methods

This retrospective, multicenter observational cohort study included patients who underwent LT for ALF between September 1991 and August 2024. The study was conducted at seven transplant centers located in Curitiba and its metropolitan region, Paraná, Brazil (Nossa Senhora das Graças Hospital, Clinical Hospital of the Federal University of Paraná, Rocio Hospital, São Vicente Hospital, Mackenzie Hospital, and Angelina Caron Hospital) and Santa Isabel Hospital, Blumenau, Santa Catarina, Brazil. Donor data were obtained from the State Transplant Centers of Paraná and Santa Catarina.

Patients younger than 12 years, those with preexisting chronic liver disease, and those with incomplete essential clinical data were excluded.

Recipient variables included demographic characteristics, ALF etiology, pretransplant clinical status, and the model for end‐stage liver disease (MELD) score, which was calculated retrospectively using the original formula based on pretransplant serum bilirubin, creatinine, and international normalized ratio (INR) values [17]. Donor variables included age, body mass index (BMI), documented infection, and history of cardiac arrest.

To evaluate the potential impact of temporal changes in organ allocation and transplant practice, transplantation era (1991–2010 vs. 2011–2024) was evaluated as a sensitivity analysis in the univariate and multivariable logistic regression models. The 2011 cutoff was chosen because it corresponded to the nationwide implementation of the Brazilian National Transplant System’s electronic organ allocation platform (SIGSNT).

The primary outcome was 30‐day mortality after LT. Vital status was ascertained from the date of transplantation until death or the last available follow‐up.

### 2.1. Statistical Analysis

Continuous variables were expressed as mean ± standard deviation (SD) or median with the corresponding measures of dispersion, as appropriate, and categorical variables as absolute numbers and percentages. Comparisons between groups were performed using Student’s *t*‐test or the Mann–Whitney *U* test for continuous variables and Pearson’s chi‐square test or Fisher’s exact test for categorical variables. A two‐sided *p* value < 0.05 was considered statistically significant.

Predictors of 30‐day mortality were evaluated using binary logistic regression. Candidate variables for the multivariable model were selected based on clinical relevance and biological plausibility rather than by automated variable selection procedures. Considering the available sample size, four clinically relevant candidate variables were included to minimize the risk of overfitting. Multicollinearity among the predictors was assessed using the variance inflation factor (VIF), and all predictors demonstrated low collinearity (all VIF values < 2.0). Missing data were handled using a complete‐case (listwise deletion) approach, and no imputation methods were applied.

Results are presented as odds ratios (ORs) with corresponding 95% confidence intervals (95% CIs). Model discrimination was assessed using the area under the receiver operating characteristic curve (AUC), and overall model fit was evaluated using Nagelkerke’s pseudo‐*R* ^2^.

The study was approved by the Institutional Review Board of Nossa Senhora das Graças Hospital, Brazil (CAAE: 85303124.4.1001.0269), and the requirement for informed consent was waived due to the retrospective design.

## 3. Results

A total of 4160 LTs were performed between September 1991 and August 2024 in the seven transplant centers.

ALF accounted for 2.43% (*n* = 101) of all LTs performed during the study period. After excluding four patients younger than 12 years, six patients with incomplete essential clinical data, and one recipient of living‐donor LT, the final study population comprised 90 recipients of deceased‐donor LT.

### 3.1. Clinical and Epidemiological Characteristics

The clinical and epidemiological characteristics of all 90 recipients are summarized in Table 1.

**TABLE 1:** Recipient characteristics and indications for LT in ALF.

**Characteristics**	**Recipients *n* = 90**

Age at transplantation (years)	
Mean ± SD	37 ± 16
Median (min–max)	35 (14–73)
Gender [*N* (%)]	
Male	23 (25.6)
Female	67 (74.4)
BMI (kg/m^2^)	*n* = 85
Mean ± SD	24.7 ± 3.9
Median (min–max)	24.2 (17.3–39.2)
Blood type [*n* (%)]	*n* = 85
A	33 (38.8)
B	14 (16.5)
AB	5 (5.9)
O	33 (38.8)
MELD score	*n* = 79
Mean ± SD	34.9 ± 5.7
Median (IQR)	37 (32–40)

**Indications for LT in ALF**	***n* = 90 (%)**

Indeterminate	27 (30)
Nonparacetamol DILI^∗^	25 (27.8)
Viral hepatitis^∗∗^	13 (14.5)
Autoimmune hepatitis	8 (8.9)
Other causes^∗∗∗^	6 (6.7)
Wilson’s disease	4 (4.4)
Paracetamol toxicity	4 (4.4)
Pregnancy‐related	3 (3.3)

*Note:* Nonparacetamol DILI^∗^: herbal products (*n* = 7); NSAIDs (*n* = 5); antibiotics (*n* = 4); halothane (*n* = 2); ART (*n* = 1); imatinib (*n* = 1); MDMA (*n* = 1); pesticides (*n* = 1); not reported (*n* = 3). Viral hepatitis^∗∗^: HBV (*n* = 7); HAV (*n* = 4); HBV reactivation (*n* = 2). Other causes^∗∗∗^: COVID‐19 (*n* = 2); dengue infection (*n* = 2); leptospirosis (*n* = 1); not reported (*n* = 1). ART, antiretroviral therapy; COVID‐19, coronavirus disease 2019; IQR, interquartile range; MDMA, 3,4‐methylenedioxymethamphetamine; NSAIDs, nonsteroidal anti‐inflammatory drugs.

Abbreviations: DILI, drug‐induced liver injury; HAV, hepatitis A virus; HBV, hepatitis B virus.

Of 90 recipients, 67 were female (74.4%), and 23 were male (25.6%), with a median age at transplantation of 35 years (range, 14–73) and a mean of 37 ± 16 years. Median BMI was 24.2 kg/m^2^ (range, 17.3–39.2; *n* = 85).

Pretransplant serum creatinine was available in 75 patients (mean 1.60 ± 1.29 mg/dL; median 1.21 [range, 0.20–6.55]). INR was available for 75 patients (mean 4.10 ± 2.41; median 3.57 [IQR, 2.49–5.01]). MELD score was available for 79 patients (mean 34.9 ± 5.7; median 37.0 [IQR, 32.0–40.0]), reflecting severe liver dysfunction at transplantation.

Renal failure occurred in 32 of 81 patients (39.5%), and 18 of them (56.3%) required dialysis (median duration, 2 days [range, 0–17]). Mechanical ventilation was required during the pretransplant period in 48 of 81 patients (59.3%), and vasoactive drug support was required in 29 of 78 patients (37.2%), indicating high preoperative severity.

The most frequent causes of ALF were nonparacetamol DILI in 25 (27.8%) patients and viral hepatitis in 13 (14.5%), as presented in Table 1. The etiology was indeterminate in 27 (30%) patients. All patients fulfilled the King’s College or Clichy criteria for urgent LT.

Of 77 donors with available gender data, 32 (41.6%) were female, and 45 (58.4%) were male, with a median age of 37 years (range, 10–75) and a mean of 38 ± 15 years (*n* = 81). Median donor BMI was 24.7 kg/m^2^ (range, 15.2–40.5; *n* = 76). Donor infection and prior cardiac arrest were reported in 14 (18.4%) and 19 (25.0%) of the 76 donors with available data, respectively.

Median hospital stay was 13 days (range, 0–80; *n* = 74), and median ICU stay was 6 days (range, 0–55; *n* = 75). Operative time was recorded in 58 patients (median 316.5 min [range, 180–535]), and cold ischemia time was recorded in 73 patients (median 360 min [range, 60–720]).

Perioperative complications were documented in 74 patients and occurred in 34 cases (46%). Bleeding was the most common complication, occurring in 16 of 34 patients (47%), followed by other miscellaneous complications in 12 of 34 patients (35.3%), whereas arterial thrombosis and rejection episodes were infrequent, occurring in 2 of 34 (6%) and 4 of 34 patients (11.7%), respectively.

Among the 44 survivors, median follow‐up was 116.5 months (range, 6–211), with a mean of 103.4 ± 54.5 months.

### 3.2. Predictors of 30‐Day Mortality

Thirty‐day mortality occurred in 46 patients (51%), while 44 (49%) survived for more than 30 days. Of the 57 total deaths observed during follow‐up, 46 (80.7%) occurred within the first 30 days, indicating that mortality was predominantly early; therefore, 30‐day mortality was defined as the primary endpoint.

In univariate and multivariable analyses, pretransplant recipient severity markers were strongly associated with 30‐day mortality, as shown in Table 2. Because of missing data, the number of patients included in each univariate analysis varied according to the availability of each variable (Table 2). Only 67 patients had complete data for all variables included in the final multivariable model and were therefore included in the complete‐case analysis.

**TABLE 2:** Logistic regression analysis for 30‐day mortality.

Variable	*n*	Univariate OR (95% CI)	*p* value	Multivariable OR (95% CI)	*p* value
Donor age ≥ 50 years	81	3.27 (1.16–10.3)	**0.031**	3.73 (0.93–17.3)	0.073
Vasoactive drug use	78	5.41 (2.01–16.1)	**0.001**	1.54 (0.33–7.36)	0.6
Mechanical ventilation	81	4.00 (1.59–10.6)	**0.004**	5.46 (1.21–28.9)	**0.033**
Serum creatinine (per 1‐mg/dL increase)	75	2.78 (1.59–5.6)	**0.001**	3.49 (1.69–8.72)	**0.003**

*Note:* Model performance: AUC = 0.831; Nagelkerke pseudo‐*R* ^2^ = 0.446. The “*n*” column indicates the number of patients with available data for each variable in the total cohort (*n* = 90). Bold values indicate statistical significance (*p* < 0.05).

Abbreviations: CI, confidence interval; OR, odds ratio.

**Figure 1:**
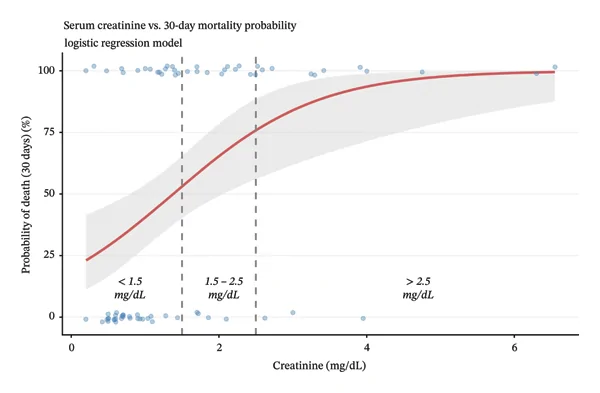
Relationship between pretransplant serum creatinine and probability of 30‐day mortality after liver transplantation. The curve demonstrates a progressive increase in mortality risk with increasing creatinine levels.

Each 1‐mg/dL increase in pretransplant serum creatinine was associated with higher odds of 30‐day mortality in both the univariate analysis (OR 2.78, 95% CI 1.59–5.60; *p* = 0.001) and the multivariable analysis (OR 3.49, 95% CI 1.69–8.72; *p* = 0.003), as illustrated in Figure 1. Renal failure was significantly associated with 30‐day mortality in univariate analyses (OR 5.00, 95% CI 1.92–14.1; *p* = 0.001).

The need for mechanical ventilation prior to transplantation was associated with higher 30‐day mortality in both univariate and multivariable analyses (OR 4.00; 95% CI 1.59–10.6; *p* = 0.004 and OR 5.46; 95% CI 1.21–28.9; *p* = 0.033, respectively). Vasoactive drug use was also associated with early mortality in univariate analyses (OR 5.41; 95% CI 2.01–16.1; *p* = 0.001), reflecting the impact of preoperative hemodynamic instability.

Intraoperative blood transfusion requirements were higher in patients who died within 30 days compared with survivors (median 4 units [1–11] vs. 2 units [0–12], *p* = 0.00756). This variable was not included in the multivariable analysis because it represented an intraoperative variable.

Donor age was evaluated using clinically relevant thresholds (≥ 50, ≥ 55, ≥ 60, and ≥ 65 years). Among the 81 recipients with available donor age information, 21 received grafts from donors aged ≥ 50 years, whereas only 7 and 3 recipients received grafts from donors aged ≥ 60 and ≥ 65 years, respectively. Because the number of recipients decreased substantially with donor age increase, the higher thresholds resulted in unstable estimates due to the limited sample size. Therefore, ≥ 50‐year threshold was retained for subsequent analyses because it included the largest number of patients while remaining clinically relevant. Thirty‐day mortality was 71.4% (15/21) among recipients of grafts from donors aged ≥ 50 years, compared with 43.3% (26/60) among recipients of grafts from donors aged < 50 years. In the univariate logistic regression analysis, donor age ≥ 50 years was associated with increased 30‐day mortality (OR 3.27, 95% CI 1.16–10.3; *p* = 0.031).

Other variables were also evaluated but showed no significant association with 30‐day mortality. Donor infection (*p* = 0.975) and donor history of cardiac arrest (*p* = 0.894) were not associated with the outcome. Similarly, recipient‐related factors, including gender (*p* = 0.185), age (*p* = 0.382), and INR (*p* = 0.619), were not significantly associated with 30‐day mortality.

As an exploratory sensitivity analysis, transplantation era (1991–2010 vs. 2011–2024) was evaluated to assess the potential impact of temporal changes on organ allocation and transplant practice. Transplantation era was not associated with 30‐day mortality (OR 2.56, 95% CI 0.04–179; *p* = 0.7). Furthermore, inclusion of this variable in the multivariable model did not materially change the estimated effects of the other variables.

**Figure 2:**
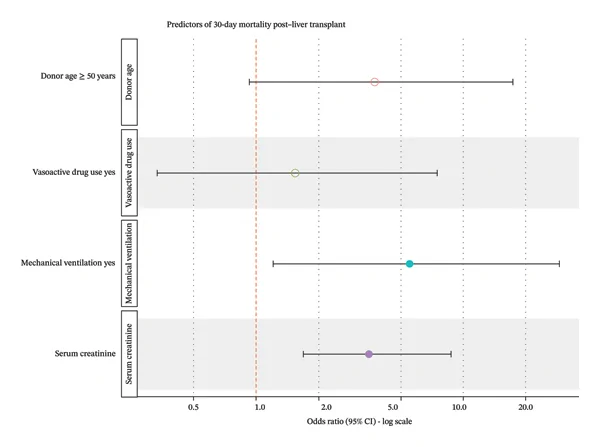
Predictors of early mortality after liver transplantation for acute liver failure. Forest plot showing the association between donor and recipient variables and the risk of 30‐day mortality after liver transplantation. Odds ratios (OR) with corresponding 95% confidence intervals (CI) were derived from logistic regression analysis. Recipient‐related variables reflecting clinical severity before transplantation included preoperative serum creatinine, need for mechanical ventilation, and vasoactive drug use, while donor‐related factors included donor age ≥ 50 years. The dashed vertical line represents the null value (OR = 1).

In the multivariable logistic regression analysis, only pretransplant serum creatinine and the need for mechanical ventilation remained independently associated with 30‐day mortality. Donor age ≥ 50 years and vasoactive drug use were associated with mortality in the univariate analysis but did not retain statistical significance after adjustment, as shown in Figure 2.

The discriminative performance of the final multivariable model was evaluated using the receiver operating characteristic (ROC) curve and demonstrated good discrimination, with an area under the curve of 0.831 (95% CI, 0.756–0.942).

## 4. Discussion

LT remains the only effective life‐saving therapy for patients with ALF who meet established criteria for urgent transplantation.

In this multicenter cohort, ALF accounted for 2.43% of all LTs performed during the study period, which is similar to other international registry data. In the United States, ALF accounts for approximately 2%–4% of liver transplant indications, according to the OPTN registry [9]. Similarly, data from the ELTR report that ALF represents approximately 2%–7% of adult LTs, although this proportion varies between countries and transplant programs [10].

The etiological distribution of ALF varies significantly across geographic regions. In North America, acetaminophen toxicity accounts for 40%–50% of cases, while approximately 12.2% are classified as indeterminate in several cohorts [8, 18, 19]. In contrast, in Europe, the predominant etiology is indeterminate or categorized as other causes (56%), followed by viral infections (19%) [10].

Data from Latin America remain limited; however, an Argentine study reported that 28% of cases were indeterminate, followed by autoimmune hepatitis (25%) and acute viral hepatitis (19%) [15]. In Brazil, data from 12 transplant centers showed that ALF accounted for 5.4% of liver transplants, with indeterminate etiology being the most frequent (34%), followed by DILI (27%) and autoimmune hepatitis (18%) [11].

In our cohort, indeterminate etiology accounted for 30% of cases, followed by nonparacetamol DILI (27.8%) and viral hepatitis (14.5%), highlighting important regional differences in the etiological profile of ALF.

Early mortality after LT for ALF remains a major clinical challenge. In our cohort, 30‐day mortality reached 51%. Notably, 80.7% of all deaths during follow‐up occurred within the first 30 days after transplantation, reinforcing that mortality after LT for ALF is predominantly an early postoperative event. Previous registry studies have reported early mortality rates ranging from 20% to 35% [10, 12], while 1‐year survival rates of 79%–84% have been reported in Europe and the United States [6]. In an exploratory analysis, transplantation era was not associated with 30‐day mortality in either the univariate or multivariable analysis and did not materially alter the associations observed for other predictors. Therefore, the higher mortality observed in our study is unlikely to be explained solely by temporal changes in transplantation practice. Instead, it is likely multifactorial, reflecting not only the advanced clinical severity of recipients but also healthcare system–related factors, including delayed referral, limited access to specialized care, and resource constraints in middle‐income settings, which have been associated with disparities in transplant outcomes [20–22].

Higher pretransplant serum creatinine levels were independently associated with death within 30 days (OR 3.49, 95% CI 1.69–8.72; *p* = 0.003). Renal failure was present in 32 of 81 patients (39.5%) in our cohort. Renal impairment is common in ALF and reflects systemic circulatory dysfunction and multiorgan failure [5, 23]. Previous studies evaluating outcomes after LT have similarly identified renal dysfunction as an important predictor of postoperative mortality and graft failure [24, 25]. This continuous association between pretransplant serum creatinine and 30‐day mortality is illustrated in Figure 1.

Mechanical ventilation prior to transplantation was also strongly associated with early mortality. In our cohort, the need for mechanical ventilation remained independently associated with 30‐day mortality (OR 5.46, 95% CI 1.21–28.9; *p* = 0.033). Mechanical ventilation usually reflects advanced hepatic encephalopathy or neurological deterioration. Previous studies have demonstrated that the need for ventilatory support prior to transplantation is associated with worse postoperative outcomes and increased perioperative mortality [26].

Similarly, the requirement for vasoactive drug support prior to transplantation reflects advanced circulatory failure and systemic inflammatory response. In our cohort, pretransplant vasoactive drug use was associated with early mortality in univariate analysis (OR 5.41, 95% CI 2.01–16.1; *p* = 0.001), further reinforcing the impact of pretransplant hemodynamic instability on early posttransplant outcomes. Circulatory dysfunction is a hallmark of severe ALF and often necessitates vasopressor therapy to maintain adequate organ perfusion [5, 27].

Donor characteristics also influenced posttransplant outcomes. In our study, donor age ≥ 50 years was associated with increased early mortality (OR 3.27, 95% CI 1.16–10.3; *p* = 0.031). Although higher donor age thresholds were also explored, the small number of recipients in these categories precluded reliable statistical estimates, supporting the use of the ≥ 50‐year threshold for the primary analysis. Increasing donor age has previously been associated with reduced graft survival and a higher risk of graft dysfunction after LT [11, 28, 29]. However, the use of extended‐criteria donors is often unavoidable in the setting of emergency transplantation for ALF because of the urgent need for organ allocation. Interestingly, intraoperative transfusion requirements were higher in patients who died within 30 days after transplantation. Patients who died received a median of 4 units of packed red blood cells compared with 2 units among survivors (*p* = 0.007). Increased transfusion requirements likely reflect severe coagulopathy, greater surgical complexity, and perioperative instability. Previous studies have also reported associations between intraoperative transfusion requirements and poorer outcomes after LT [30].

These findings suggest that recipient clinical severity appears to play the predominant role in determining early mortality after LT for ALF, although donor age may also contribute to postoperative risk. The final multivariable model demonstrated good discriminative performance (AUC = 0.831; 95% CI, 0.756–0.942), indicating that these readily available pretransplant variables may help identify patients at increased risk of early mortality. Nevertheless, external validation is required before these findings can be translated into clinical practice.

This study has several limitations. Its retrospective design may have introduced information bias and limited control over potential confounding factors. In addition, the study spans more than 3 decades, during which advances in surgical techniques, perioperative care, and donor selection may have influenced outcomes. However, transplantation era was not associated with 30‐day mortality in our exploratory analysis. Because of missing data, the final multivariable model was based on 67 complete cases. Despite the multicenter design, the relatively small sample size and number of events further constrained the complexity of the multivariable model. Finally, the evaluation of multiple donor age thresholds was exploratory and may have increased the risk of Type I error. These limitations should be considered when interpreting the findings.

Despite these limitations, this study provides relevant data on LT for ALF in a region where evidence remains scarce. This is one of the largest multicenter analyses evaluating predictors of early posttransplant mortality in ALF in Latin America. The inclusion of multiple transplant centers and a long observation period offers a comprehensive overview of real‐world clinical practice in this setting. Our findings emphasize the impact of recipient clinical severity and donor characteristics on early posttransplant outcomes.

In conclusion, early mortality after LT for ALF remains substantial. Higher pretransplant serum creatinine levels and the need for mechanical ventilation were independently associated with 30‐day mortality, whereas vasoactive drug support and donor age ≥ 50 years were associated with mortality in univariate analyses. These findings underscore the predominant influence of recipient clinical status on early posttransplant outcomes while suggesting that donor age may also contribute to risk. Improved pretransplant risk stratification may help optimize clinical decision‐making and perioperative management in this high‐risk population.

## Funding

No specific funding was received for this study.

## Conflicts of Interest

The authors declare no conflicts of interest.

## Data Availability

The data that support the findings of this study are available on request from the corresponding author. The data are not publicly available due to privacy or ethical restrictions.
